# Deep-subwavelength resolution detection of polar magnetization by optical spin meron lattices on hyperbolic metamaterials

**DOI:** 10.1515/nanoph-2025-0424

**Published:** 2025-10-31

**Authors:** Jingya Wu, Weiyu Wei, Kefeng Guo, Xiangyang Xie, Aiping Yang, Xinrui Lei, Peng Shi, Qiwen Zhan, Xiaocong Yuan

**Affiliations:** Nanophotonics Research Center, Shenzhen Key Laboratory of Micro-Scale Optical Information Technology & Institute of Microscale Optoelectronics, 47890 Shenzhen University, Shenzhen 518060, China; School of Optical-Electrical and Computer Engineering, University of Shanghai for Science and Technology, Shanghai 200093, China

**Keywords:** optical topological quasiparticles, spin-orbit couplings, magnetic detection, hyperbolic metamaterials, polar magnetization

## Abstract

Magnetic-optical Kerr or Faraday effects have been widely used to measure magnetic domain structures by analyzing far-field polarization properties, with resolution limited by the wavelength scale of light. Here, we propose a methodology to measure the magnetic domain at a deep-subwavelength scale by investigating the interactions between a magnetic film and a topological meron spin lattice on the surface of hyperbolic metamaterials (HMMs), which support high-**k** modes. By introducing a grating structure on the HMM surface to excite volume plasmon polaritons, optical meron spin lattices are formed on the outer surface of the HMM. Subsequently, utilizing the spin–orbit couplings of the topological lattices in the presence of magnetization, a 0.158*λ* resolution and 100 % high-precision detection of the magnetic domain structures with random polar orientations was achieved by altering the incident polarizations from right-handed to left-handed circular polarizations and summing the out-of-plane spin distributions. The findings offer opportunities for the visualization of magnetic domain structure with polar orientation of magnetization and in turn for the development of novel photonic spin topologies using complex magnetization patterns.

## 1 Introduction

Skyrmion is a type of topological quasiparticle which was first predicted in high-energy physics field by particle physicist Tony Skyrme [1]. Since then, skyrmions have been discovered in diverse physical fields, including nucleons [2], Bose–Einstein condensates [3], liquid crystals [4], magnetic materials [5], and twistronics [6]. Recently, the skyrmionic topologies have been constructed by the vector structures of diversified degrees-of-freedom of light [7], [8], [9], for example, electromagnetic (EM) field vectors [10], [11], [12], [13], [14], spatiotemporal structure [15], [16], [17], Stokes polarized vector on Poincaré sphere [18], [19], [20], [21], [22], [23], [24], [25], [26], [27], spin vectors [28], [29], [30], [31], [32], [33], [34], [35], [36], [37], [38], [39], [40], and Poynting vectors [41], and verified experimentally. Meanwhile, the optical skyrmions exhibit strong topological robustness against perturbations [42], [43], [44], [45], and this characteristic endows them with inherent anti-interference capabilities in light–matter interactions based physics and applications.

Among them, the photonic skyrmions formed by the vector structures of spin angular momentum (SAM) of surface plasmon polariton (SPP) modes at the optical near-field have been demonstrated potentially in the applications of ultrafast subwavelength imaging [30], picometer metrology [46], [47], super-resolution magnetic detection [48], and light–matter interactions at deep subwavelength scale [47], [49], [50].

In magnetic materials, various magneto-optical effects, such as the Kerr and Faraday effects, have been commonly used to measure magnetic domain structures by analyzing polarization changes in reflected or transmitted light [51], [52], [53], [54], [55]. Conventional far-field measurements are limited by the diffraction limit of light, whereas in the near field, magnetic domain structures will induce the coupling between different polarization components. By exploiting the subwavelength property of photonic topological spin textures constructed by SPPs, it becomes feasible to achieve visualization and detection of magnetic domain structures beyond the optical diffraction limit [28], [37]. Although the feature size of fine spin structure in photonic skyrmions is on the order of several tens of nanometers (nm) [47], the resolution of magnetic detection is ultimately governed by the unit cell size of the spin lattices. As the wavelength of the excited transverse magnetic (TM) EM field is 633 nm (which is also used throughout our work), the detection resolution is fundamentally limited to approximately 120 nm [48].

Hyperbolic metamaterials (HMMs) offer a promising approach to further overcome the resolution limit in magnetic domain detection [56], [57], [58], [59]. Metamaterials are artificially engineered structures with tunable permittivity tailored through their composition and periodicity, enabling exotic EM properties not found in natural materials. Among the various metamaterials, HMMs have attracted extensive interest for their extreme anisotropy, which originates from the opposite signs of the real parts of the permittivity tensor components. The hyperbolic dispersion enables the propagation of exceptionally large wavevector **k** within the material, facilitating compact control over the spin structure of the surface EM field [60]. Therefore, the resolution of magnetic detection using photonic skyrmions can be further enhanced by using HMMs, whose dielectric parameters can be tailored through careful engineering of their structural composition.

**Figure 1:**
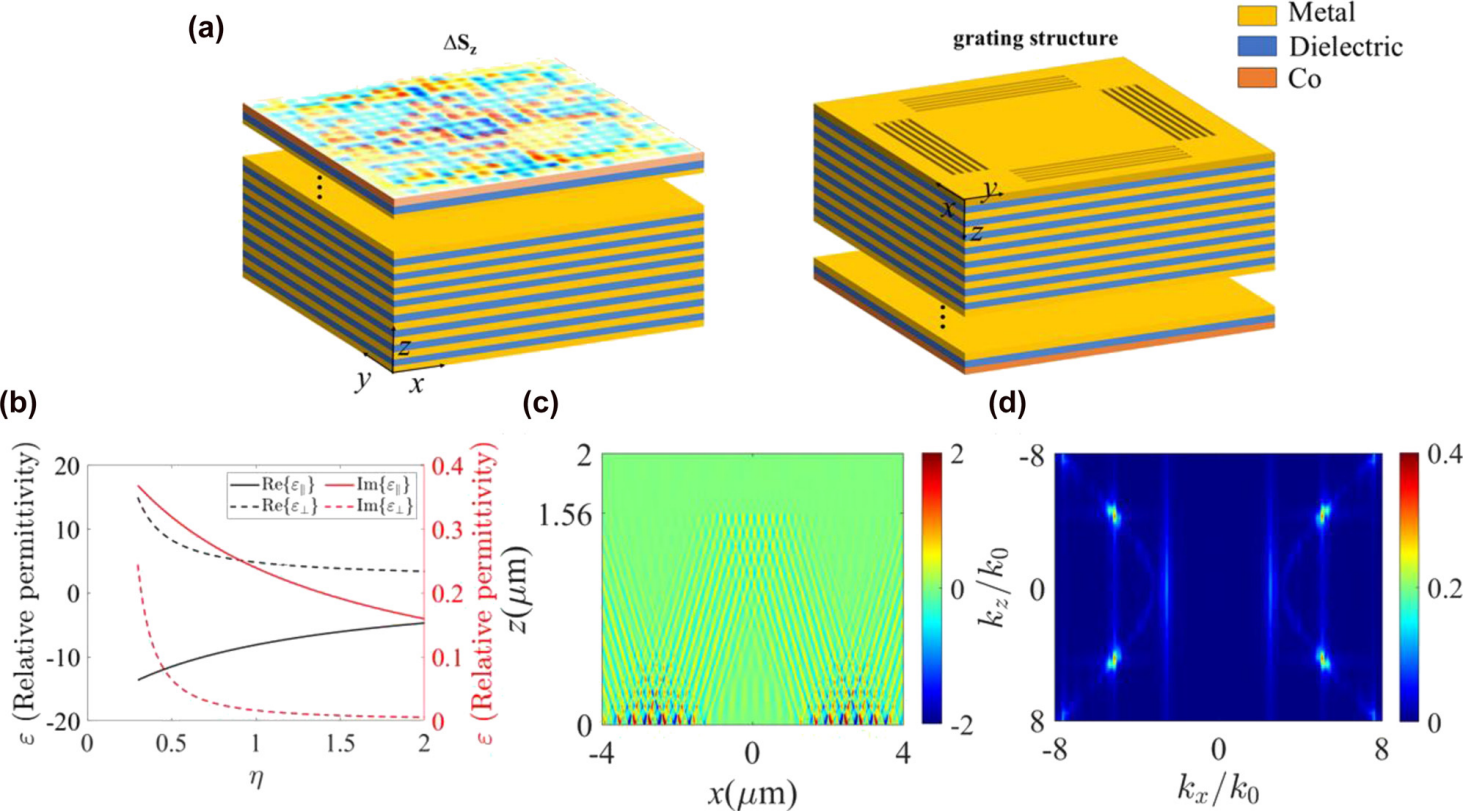
Dispersion relations in HMM-based topological meron lattices. (a) Top view and bottom view of the topological meron lattices at an HMM surface (light incident in the +*z*-direction); (b) relationship between the horizontal and vertical components of relative permittivity *ε* and the filling rate *η* (*η* varies from 0.30 to 2.0). When the filling ratio *η* ≥ 0.30, the signs of the horizontal and vertical components of the HMM’s permittivity tensor are opposite, resulting in a hyperbolic dispersion profile. When *η* = 1.4, the simulated (c) Re{*E*_*z*_} in the *xz*-plane; (d) dispersion curve obtained from the Fourier transform of the Re{*E*_*z*_}. It can be observed in (d) that the hyperbolic dispersion appears, and the vertical lines correspond to the EM modes excited by the grating. The permittivity of Ag and SiO_2_ is obtained from the experimental data as *ε*_m_ = −18.30 + 0.48*i* and *ε*_d_ = 2.12 [61].

In this work, we propose a methodology to measure the magnetic domain at a deep-subwavelength scale by investigating the interactions between a magnetic film and a topological meron spin lattice on the HMM surface. The schematic of the proposed structure is shown in Figure 1(a), where an HMM composed of silver (Ag) film and silicon dioxide (SiO_2_) film with a filling rate of 1.4 is coated on the magnetic film. By analyzing the EM properties and SAM densities on the HMM surface, we utilize the grating structures to excite the TM SPP modes at the surface of HMMs containing the hyperbolic dispersion properties to form the spin meron lattices with tunable deep subwavelength feature size. The theoretical simulations demonstrate that the spin meron lattices have a half period of 100 nm. By using these well-established spin structures, we further investigate the near-field magneto-optical interactions between the spin meron lattices and cobalt (Co) metal. When magnetization in the out-of-plane direction is present in the Co metal with a proper feature size, the sum of spin vectors with opposite chirality corresponds to the prescribed magnetization direction, achieving ultrahigh precision (100 %) recognition of the out-of-plane magnetization with a resolution of 100 nm. The findings demonstrate a feasible method for visualizing magnetic domain structures and offer possibility in the detection of magnetic domain structures at tens of nanometers by engineering the operating wavelength and the material composition and periodicity of HMMs.

## 2 Optical spin merons lattice in HMM

HMMs are artificially engineered composites typically consisting of alternating subwavelength layers of metallic and dielectric materials, or arrays of metallic nanowires embedded in a dielectric matrix, giving rise to highly anisotropic EM responses and allowing for a hyperbolic dispersion iso-frequency contour. For a uniaxial crystalline material, the permittivity tensor is typically represented by $\hat{\varepsilon }=\mathrm{diag}\left(\varepsilon_{\|},\varepsilon_{\|},\varepsilon_{⊥}\right)$ . In HMMs, the real parts of *ɛ*_‖_ and *ɛ*_⊥_ exhibit opposite signs, which underpin the hyperbolic dispersion. We consider an HMM composed of alternating layers of Ag and SiO_2_. According to the effective medium theory, a multilayered structure can be treated as a homogeneous medium when the periodicity is much smaller than the operating wavelength where the horizontal and vertical components of relative permittivity of HMMs can be expressed as [56], [57], [58], [59], [60]

(1) $$\begin{array}{c} \varepsilon_{\|}=\frac{\varepsilon_{m}+\eta \varepsilon_{d}}{1+\eta }\varepsilon_{⊥}=\frac{\varepsilon_{m}\varepsilon_{d}\left(1+\eta \right)}{\varepsilon_{d}+\varepsilon_{m}\eta } \end{array},$$

where *ε*_*m*_ and *ε*_*d*_ denote the relative permittivity of the Ag and SiO_2_ film, respectively, *η* signifies the filling rate given by the ratio of the thickness of the SiO_2_ layer to the thickness of the Ag layer.

The effective permittivity tensor of the HMM is calculated for a filling rate *η* ranging from 0.30 to 2 for a wavelength of 633 nm, as shown in Figure 1(b). When the filling ratio *η* ≥ 0.30, the signs of the horizontal and vertical components of the HMM’s permittivity tensor are opposite, resulting in a hyperbolic dispersion profile.

For a given wavelength in free space, the dispersion relation in HMMs can be expressed as [56], [57], [58], [59], [60]

(2) $$\frac{k_{t}^{2}}{\varepsilon_{⊥}}+\frac{k_{z}^{2}}{\varepsilon_{\|}}=k_{0}^{2},$$

where, *k*_*t*_ and *k*_*z*_ represent the transverse and longitudinal components of the wavevector, respectively, with $k_{t}^{2}=k_{x}^{2}+k_{y}^{2}$ , and *k*_0_ denotes the wave number of the incident light in free space. The hyperbolic dispersion relation allows modes with ultra-large wavevectors to propagate in HMMs. The direction of EM field propagation is defined by the direction of energy flow, which is given by the Poynting vector [60]. For hyperbolic dispersion with large wavevectors, the relationship between the propagation direction and the angle with respect to the optical axis can be given by [60]

(3) $$\theta =\arctan\left(\frac{\varepsilon_{\|}k_{t}}{\varepsilon_{⊥}k_{z}}\right).$$

The permittivity tensors of the HMM depend on the filling rate *η*, where the propagation angle for *η* = 1.4 is 58°.

A schematic diagram of the HMM structure is illustrated in Figure 1(a). The thicknesses of Ag and SiO_2_ layers are 10 nm and 14 nm, respectively. The metamaterial is treated as a uniaxial crystal with a total thickness of 1.56 μm and an effective permittivity tensor of *ε*_HMM_ = diag(−6.38 + 0.20*i*, −6.38 + 0.20*i*, 3.97 + 0.009*i*). The finite-difference time-domain (FDTD) method is used to demonstrate the EM properties and spin topology of the surface wave sustained at the air/HMM interface. The simulation region in the *xy*-plane is 4.0 μm × 4.0 μm. A 50 nm silver layer is coated on the top surface of the HMM, and a finite-sized grating structure with air grooves arranged periodically in four directions is used to excite the VPP modes. The grating period is set to 400 nm, with filling rate *f* = 0.5, and the individual grating size is 2.8 μm × 2.0 μm. The focused circularly polarized Gaussian beam is used to illuminate the grating structure, introducing phase delays among the four interfering plasmonic fields. The simulated longitudinal electric field component distribution *E*_*z*_ along the *xz*-plane for *η* = 1.4 is shown in Figure 1(c). The optical field excited by the grating structure forms an evanescent field within a 1.9 μm × 1.9 μm region at the center of the HMM. The in-plane propagation constant of the VPPs excited by the grating is determined by the grating equation. Since $k_{t}^{\prime }<k_{0}\sqrt{\varepsilon_{⊥}}$ for *m* = 1, the condition is only satisfied for *m* = 2, 3, …. The 2-order mode (*m* = 2) can be excited by the spatial period of *λ*_*d*_ = 200 nm. The frequency domain of the longitudinal electric field component is illustrated in Figure 1(d), with the vertical lines corresponding to EM modes. The first-order EM mode does not intersect with the hyperbolic modes, and thus the intersection between the dispersion curve of the second-order mode and that of the hyperbolic modes corresponds to the EM field modes supported by the HMM.

The magnitude of the spin vector **S** of the optical field is defined as [62]

(4) $$S=\frac{1}{4\omega }\text{Im}\left\{\varepsilon_{0}E^{*}\times E+\mu_{0}H^{*}\times H\right\},$$

where *ω* represents the angular frequency of the incident light, while *ε*_0_ and *μ*_0_ denote the permittivity and permeability of vacuum, respectively. Because most of near-field microscopic techniques measure the electric components of EM field (**E**) [63], we only investigate the electric part of the spin vector Im{**E** ^*^ × **E**} so that the spin vector can be re-expressed by **S** = *A*Im{**E** ^*^ × **E**}, where *A* is a constant.

For the surface waves at the metal/air interface, the breaking of continuous rotational symmetry in an individual photonic spin skyrmion and the transition from continuous to discrete rotational symmetry will result in the formation of photonic topological lattices, with the spin topology determined by the symmetry of EM fields [28]. Under 4-fold symmetry, photonic spin meron lattices with alternating skyrmion numbers *Q* = ±1/2 in each unit cell are formed, arising from the conservation of total angular momentum and spin–orbit couplings [31], [32]. This principle also applies to surface waves generated at the HMM/air interface. In the HMM structure, the SAM densities can be expressed as

(5) $$\left\{\begin{array}{c} S_{x}=\frac{\varepsilon_{\|}\beta }{k_{z}\varepsilon_{⊥}}{\left|A\right|}^{2}\sin\left(\beta x\right)\cos\left(\beta y\right),\\ S_{y}=\frac{\varepsilon_{\|}\beta }{k_{z}\varepsilon_{⊥}}{\left|A\right|}^{2}\cos\left(\beta x\right)\sin\left(\beta y\right),\\ S_{z}={\left|A\right|}^{2}\cos\left(\beta x\right)\cos\left(\beta y\right), \end{array}\right.$$

for the quantum number of total angular momentum *l* = 1, where *β* is the propagation constant. In HMMs, the hyperbolic dispersion characteristics can be exploited to control the propagation constant of the surface wave (effective index of surface wave), thereby enabling precise control over the spatial scale of photonic spin meron lattices. Previously, researchers proposed a deep subwavelength interference lithography method based on volume plasmon polaritons (VPPs) with tunable pattern periodicity [64]. Here, we use a similar metal/air grating method to excite the VPPs in HMMs, where the in-plane propagation constant of the VPPs should satisfy the grating equation. For normal incident light, this equation is given by $k_{t}^{\prime }=2m\pi /d$ , where *d* is the grating constant and *m* is an integer. The hyperbolic dispersion of HMMs supports the existence of high-**k** wavevector modes, with stable propagation modes forming only when $k_{t}^{\prime }>k_{0}\sqrt{\varepsilon_{⊥}}$ . Since $k_{t}^{\prime }>k_{0}$ , a wave vector mismatch arises for VPPs at the air/HMM interface, preventing the propagation of VPPs modes. Consequently, VPPs modes are transformed into evanescent wave modes. When these evanescent waves satisfy the symmetry conditions, the spin structure exhibits periodicity.

**Figure 2:**
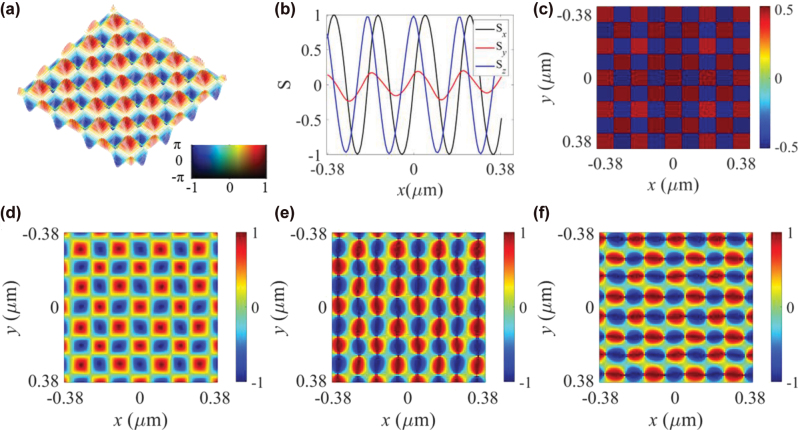
Formation of optical meron lattices at the air/HMM interface. (a) The distribution of spin vector for photonic spin meron lattice when the quantum number of total angular momentum *l* = 1 (i.e., incident light is the right-handed circularly polarized light), (b) the one-dimensional (1D) contour of SAM density along the *x*-axis, and (c) the calculated skyrmion number *Q* for each unit cell. The calculated skyrmion number is approximately binary since *Q* ≈ ±1/2 for each unit cell. The simulated distributions of (d) *S*_*z*_, (e) *S*_*x*_, and (f) *S*_*y*_.

Subsequently, Figure 2(a) illustrates the calculated distribution of the normalized longitudinal SAM vector, with the unit cell size of the optical spin meron lattice given by *λ*_*l*_ = *λ*_*d*_/2 = 100 nm, approximately 0.158*λ*_0_. A cross-sectional view of the SAM vector along the *x*-axis is depicted in Figure 2(b), demonstrating the deep-subwavelength scale feature of photonic topological lattices. The skyrmion number for a single unit cell is defined as $Q=1/4\pi \int \int n\cdot \left(\partial_{x}n\times \partial_{y}n\right)\text{d}x\text{d}y$ [5], where **n** = **S**/|**S**|. The FDTD simulations show that the skyrmion number *Q* is approximately +0.5 for unit cells with the spin vector in the “upward” state and −0.5 for those in the “downward” state, as shown in Figure 2(c). When altering the incident light from left-handed to right-handed circularly polarized light, due to the spin-momentum locking relationship of optical transverse spin for surface wave, the resulted spin meron lattice exhibit opposite spin states, i.e., the unit cell at the same zone has an opposite skyrmion number.

## 3 Near-field magneto-optical interactions

When a magnetic material is introduced, the interaction between the electric field and spin-polarized electrons gives rise to the magneto-optical effect, resulting in off-diagonal components in the material’s permittivity tensor. For polar magnetization along the *z*-axis, the permittivity tensor is given by [48]

(6) $${\hat{\varepsilon }}_{\text{MO}\;}=\left(\begin{array}{ccc} \varepsilon_{i} & -ig_{z} & 0\\ ig_{z} & \varepsilon_{i} & 0\\ 0 & 0 & \varepsilon_{i} \end{array}\right),$$

where *g*_*z*_ is the gyration vector constant that is linear in magnetization. The presence of the off-diagonal gyration term *g*_*z*_ would lead to the couplings between the TM and transverse electric (TE) modes, enabling hybridization of the two polarizations and mode conversion. By applying Maxwell’s equations, the wave equation inside a magnetic layer is derived as

(7) $$\nabla \times \nabla \times E=k_{0}^{2}{\hat{\varepsilon }}_{\text{MO}}E.$$

In Eq. (7), the wavevector for the TM mode is approximated as $k_{n\mathrm{zi}}^{\pm }\text{≈}k_{\mathrm{zi}}^{0}\pm \;\Delta k,$ where $k_{\mathrm{zi}}^{0}=\surd \left(k_{t}^{2},-,\varepsilon_{i},k_{2}^{0}\right)\;\text{and}\;\Delta k=ik_{0}|g_{z}|/\left(2,\surd ,\left(\varepsilon_{i}\right)\right)$ .

To investigate the EM properties inside the magnetic layer, a 4 × 4 transfer matrix method is used. Considering the SPPs at an interface between a dielectric layer (layer 1) and a magnetic layer (layer 2), the ratio between the electric field components *E*_*y*_ and *E*_*x*_ is given by [48]

(8) $$\eta_{z}=-\frac{i\varepsilon_{1}^{2}k_{0}^{2}}{2\left(\varepsilon_{1}-\varepsilon_{2}\right)\varepsilon_{2}k_{z1}^{2}}g_{z},$$

where *ε*_1_ and *ε*_2_ are the dielectric constants of the dielectric layer and the magnetic material layer, respectively, and $k_{z1}=\sqrt{k_{t}^{2}-\varepsilon_{1}k_{0}^{2}}$ .

**Figure 3:**
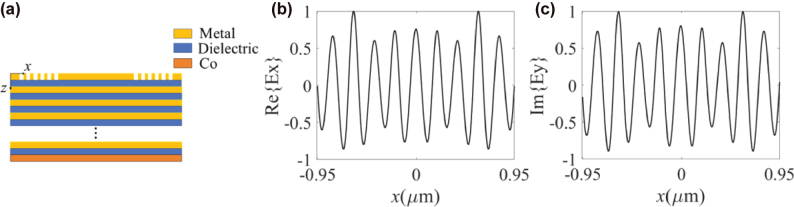
Cross-sectional view of the HMM structure and corresponding electric field distributions. (a) Cross-sectional view of the magnetized cobalt (Co) thin film placed below the HMM structure; (b) real part of *E*_*x*_ along the *x*-axis and (c) imaginary part of *E*_*y*_ along the *x*-axis excited by an *x*-polarized incident light. The intensities in (b) and (c) are normalized by the maximal values of the corresponding field components.

In the HMM multilayer structure shown in Figure 1(a), a grating with periodic arrangement along the *x*-direction and infinite length along the *y*-direction is considered (Figure 3(a)). A 50 nm Co magnetic layer with magnetization oriented along the +*z*-direction is placed behind the HMM. When *x*-polarized plane wave is illuminated onto the HMM structure from the top side, the simulated electric field at the Co/air interface is shown in Figure 3(b) and (c). The real part of the electric field, Re{*E*_*x*_}, originates from the grating excitation, whereas the imaginary part, Im{*E*_*y*_}, results from the magneto-optical coupling between different polarization components in the Co layer. Since *η*_*z*_ is approximatively purely imaginary when the loss is negligible, the imaginary part of the coupled electric field component is dominated, corresponding to a phase shift of *π*/2 between *E*_*x*_ and *E_y_*. Here, *η*_*z*_ ≈ 0.024*i*. Due to the coupling of magnetization to TE polarization, the meron lattice is modulated by the gyration vector *g*_*z*_. When the incident light is circularly polarized (CP), by neglecting the ultrasmall oscillatory, attenuation, and asymmetric terms, the *z*-component of the SAM density can be given by

(9) $$\begin{array}{ll} S_{z}^{\text{CP}} & =A\text{Im}\;\left\{E^{*}\times E\right\}=A\left[\text{Im}\;\left\{\left(E_{x}^{*}E_{y}+E_{y}^{*}E_{x}\right)\right\}\right.\\ & \;\times \left.\left(1-{\left|\eta_{z}\right|}^{2}\right)+2\left({\left|E_{x}\right|}^{2}+{\left|E_{y}\right|}^{2}\right)\text{Im}\;\left\{\eta_{z}\right\}\right]. \end{array}$$

From Eq. (9), the first term on the right-hand side is determined by the chirality of incident CP light, and thus this term can be canceled by the superposition of spin vectors generated by the right-handed CP (RCP) light and left-handed CP (LCP) light. The second term on the right-hand side is related to the magnetization and the intensity of the in-plane electric field. Moreover, the variation of *z*-component SAM density Δ*S*_*z*_ is proportional to the imaginary part of *η*_*z*_, where *η*_*z*_ is purely imaginary, and the magnetization direction determines the sign of Δ*S*_*z*_. Consequently, the relation shown in the second term on the right-hand side of Eq. (9) between Δ*S*_*z*_ and the magnetization direction can be utilized to detect the out-of-plane magnetization orientation. By importing the LCP and RCP light alternately, spin meron lattices with opposite topological skyrmion number can be constructed. The SAM densities are modulated by the *z*-direction magnetization of the Co material, and a near-field scanning imaging technique can scatter the near-field SPP electric field to the far-field via well-designed nanoparticles [63]. A low numerical aperture (NA) objective lens then acts as a low-pass filter to collect the in-plane electric field component of the radiation field. After measuring the in-plane RCP and LCP components with this near-field scanning imaging techniques, *S*_*z*_ can be obtained [63]. Finally, this variation can be used to characterize the magnetic domain structure, and in particular, the small scale and configuration of photonic meron lattices can achieve higher precision and flexible detection of arbitrary magnetic domain structures.

## 4 Results

A 50 nm thick Co metal layer is placed behind the HMM, with *ε*_2_ = −12.50 + 18.46*i* [65] and *g*_*z*_ = 0.59−0.48*i* [66]. The magnetization direction is set to ±*z*, with the dimension of each magnetic domain unit corresponding to that of the unit cell of the meron lattices. In this instance, the sum of *S*_*z*_ values at the same position for incident LCP and RCP light does not fully cancel out. The air/silver slit structure of the excited grating acts as a linear dipole. Its size, comparable to the light field, introduces non-negligible asymmetry in the excited electric field. For a finite-length linear dipole aligned along the *x*-axis with its dipole moment along the *y*-axis, the *y*-component of the electric field exhibits opposite signs but equal magnitudes at symmetric points along the *x*-axis. This asymmetry is also manifested in *S*_*x*_ and *S*_*y*_. To eliminate this effect, the sum $\hat{T}\left(S_{z}^{\text{LCP}}\right)+S_{z}^{\text{RCP}}$ instead of $S_{z}^{\text{LCP}}+S_{z}^{\text{RCP}}$ is used to measure the polar magnetization of the Co material, where $\hat{T}$ denotes the transpose of matrix.

In addition, due to the breaking of optical field symmetry caused by the finite-length grating structure, the directly measured Δ*S*_*z*_ cannot accurately reconstruct the distribution of magnetic domain. Therefore, only if the magnetic domain distribution remains identical after the transpose operation, i.e., the random magnetization matrices are symmetric, we can use this operation ( $\Delta S_{z}=\hat{T}\left(S_{z}^{\text{LCP}}\right)+S_{z}^{\text{RCP}}$ ) to achieve the high-precision measurement of polar magnetization.

**Figure 4:**
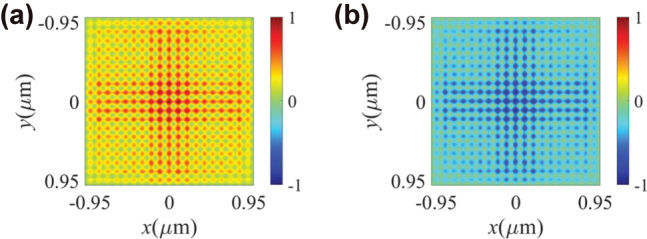
Simulated variation of *z*-component SAM density Δ*S*_*z*_ for the opposite magnetization direction. (a) The simulation results Δ*S*_*z*_ for the magnetization direction set to +*z* and (b) the simulation results for Δ*S*_*z*_ for the magnetization direction set to −*z*. The simulation results are normalized by the maximal absolute values of the corresponding Δ*S*_*z*_.

When the magnetization of Co is uniformly oriented along ±*z* directions, the simulated Δ*S*_*z*_ distributions reflect the magnetic domain through corresponding changes in sign, as shown in Figure 4. In Figure 4(a), the magnetization direction of Co material is +*z*, while in Figure 4(b), the magnetization direction is −*z*, resulting in Δ*S*_*z*_ > 0 and Δ*S*_*z*_ < 0, respectively, which effectively corresponds to the polar magnetization direction of Co.

**Figure 5:**
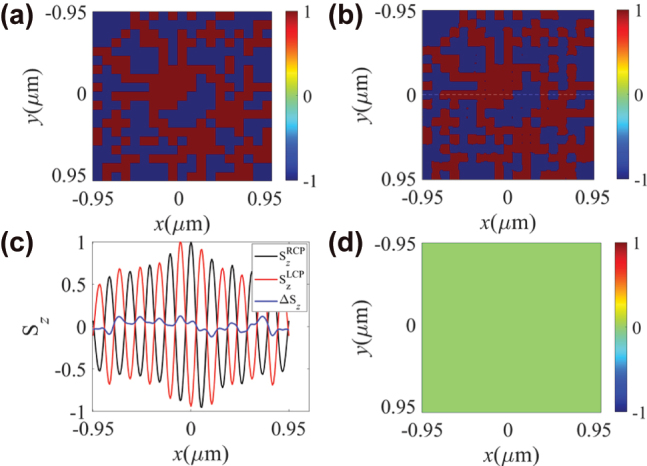
Results and errors of the magnetic detection. (a) A random magnetic domain matrix with magnetization along ±*z*-direction; (b) binarized summation result of Δ*S*_*z*_ within unit cells; (c) *S*_*z*_ and Δ*S*_*z*_ along the white dashed line in the *x*-direction; (d) error of binarized summation of Δ*S*_*z*_ within unit cells. All the simulated results are normalized by the corresponding maximal values.

**Figure 6:**
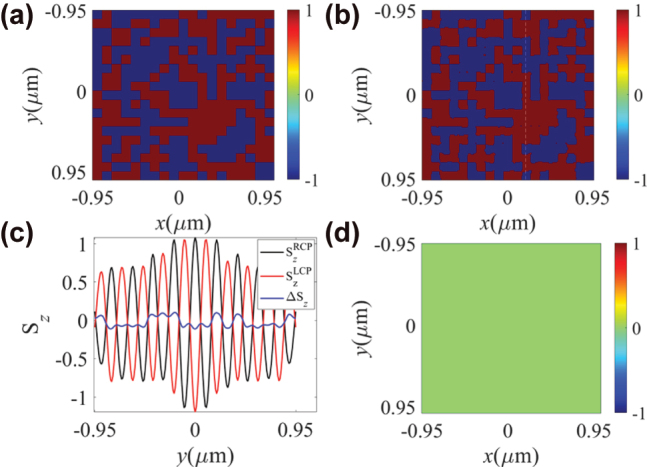
Results and errors of the magnetic detection. (a) A random magnetic domain matrix with magnetization along the ±*z*-direction; (b) binarized summation result of Δ*S*_*z*_ within unit cells; (c) *S*_*z*_ and Δ*S*_*z*_ along the white dashed line in the *y*-direction; (d) error of binarized summation of Δ*S*_*z*_ within unit cells. All simulated results are normalized by the corresponding maximal values.

Based on these analyses, further simulations were performed using a random ±*z*-direction magnetic domain array. The randomly distributed *z*-direction magnetization matrix set in the Co layer is shown in Figures 5(a) and 6(a). Figure 5(b) exhibits the binarized result of Δ*S*_*z*_, where Δ*S*_*z*_ is binarized to ±1 based on its sign.

The spatial distribution of the binarized Δ*S*_*z*_ in Figure 5(b) is consistent with the preset magnetic domain with the random *z*-direction distribution of magnetization as shown in Figure 5(a). The absolute value of *S*_*z*_ decreases as the observation point is away from the center, and Δ*S*_*z*_ is proportional to the intensity of the in-plane electric field. Consequently, the unit cells at the edges are more sensitive to noise compared to those at the center, resulting in a reduction of the reliability of the measured results. Figure 5(c) illustrates the spatial variation of non-binarized $S_{z}^{\text{LCP}}$ , $S_{z}^{\text{RCP}}$ , and Δ*S*_*z*_ along the white dashed line in Figure 5(b), with a spatial resolution of the magnetic domains along the *x*-axis of 100 nm (full width at half maximum (FWHM)). Additionally, since *S*_*z*_ exhibits periodic spatial variation, there are zero points where *S*_*z*_ = 0. The zero points, located at the boundaries of unit cells, are more prone to deviations compared to the extremum points of *S*_*z*_ at the center of the unit cells. Therefore, here we propose an integration method by summing the data of an entire unit cell as the response of this unit cell to enhance the detection precision. The result of subtracting the integrated response from the set random magnetic domain distribution is shown in Figure 5(d), which indicates the precision of magnetic domain detection is 100 %.

From Figures 5(d) and 6(d), it can be observed that the detected results using the integrated method show no discrepancy with the pre-set distributions of the magnetic domain matrix, indicating that the primary component of Δ*S*_*z*_ within a single unit cell exhibits no significant deviation. Therefore, we can conclude that the Δ*S*_*z*_ results can accurately map the positive and negative magnetization directions along the *z*-axis. Similarly, Figure 6(d) shows the spatial variation of $S_{z}^{\text{LCP}}$ , $S_{z}^{\text{RCP}}$ , and Δ*S*_*z*_ along the white dashed line in Figure 6(b) with respect to the *y*-axis, with a spatial resolution of the magnetic domains of 100 nm (FWHM). The detection resolution is determined by the unit cell size of the optical spin meron structure, and thus it can be enhanced further by engineering the operating wavelength or the material composition and periodicity of HMMs.

## 5 Conclusions

We investigate the optical topological spin structures in HMMs and their interactions with the magnetization directions of magnetic metal materials. Through theoretical calculations, the characteristics of the spin vector field of the plasmonic field formed on the HMM surface were analyzed in detail. A method for detecting magnetization directions based on photonic spin meron structures was proposed. By introducing a grating structure on the top of HMM to excite the VPPs, photonic spin meron lattices were formed on the surface of the HMM. Utilizing the structures for magnetization directions, high-resolution detection (100 %) of the polar magnetization of magnetic domains was achieved by measuring the variation in Δ*S*_*z*_. Simulation results under specific parameter settings demonstrated the ability to detect the *z*-direction orientation of individual magnetic domains with dimensions of 100 nm × 100 nm and a total measurement range of 1.9 μm × 1.9 μm. The resolution and measurement range can be further improved by designing the nanoprobe based on the HMM structure. Our findings provide a novel and effective pathway for high-precision magnetic domain detection with deep subwavelength resolution.
